# testCompareR: an R package to compare two binary diagnostic tests using paired data

**DOI:** 10.12688/wellcomeopenres.22411.3

**Published:** 2024-10-21

**Authors:** Kyle J. Wilson, José A. Roldán-Nofuentes, Marc Y.R. Henrion

**Affiliations:** 1 University of Liverpool, Liverpool, L7 8TX, UK; 2 Malawi-Liverpool-Wellcome Trust Clinical Research Programme, Blantyre, Southern Region, Malawi; 3 Universidad de Granada, Granada, Andalusia, 18010, Spain; 4 Liverpool School of Tropical Medicine, Liverpool, L3 5QA, UK

**Keywords:** R package, diagnostic test, paired data, dichotomous, binary, compare

## Abstract

**Background:**

Binary diagnostic tests are commonly used in medicine to answer a question about a patient’s clinical status, most commonly, do they or do they not have some disease. Recent advances in statistical methodologies for performing inferential statistics to compare commonly used test metrics for two diagnostic tests have not yet been implemented in a robust statistical package.

**Methods:**

Up-to-date statistical methods to compare the test metrics achieved by two binary diagnostic tests are implemented in the new R package testCompareR. The output and efficiency of testCompareR is compared to the only other available package which performs this function, DTComPair, as well as an open-source program, compbdt, using a motivating example.

**Results:**

testCompareR achieves similar results to DTComPair using statistical methods with improved coverage and asymptotic performance. Further, testCompareR is faster than the currently available package and requires fewer pre-processing steps in order to produce accurate results.

**Conclusions:**

testCompareR provides a new tool to compare the test metrics for two binary diagnostic tests compared with the gold standard. This tool allows flexible inputs, which minimises the need for data pre-processing, and operates in very few steps, so that it is easy to use even for those less experienced with R. testCompareR achieves results comparable to those computed by DTComPair, using optimised statistical methods and with improved computational efficiency.

## Plain Language Summary

testCompareR is a new package for the statistical programming language R which compares the performance of two binary diagnostic tests. Binary diagnostic tests are tests which give either a positive or negative outcome. A good example is the lateral flow test commonly used to diagnose COVID-19, but they are widely used in medicine.

testCompareR is faster and more efficient than existing options like DTComPair, with comparable accuracy and fewer pre-processing requirements. This means it's easier to use, especially for those new to R. testCompareR is a valuable option for researchers and clinicians needing to compare test metrics from two binary diagnostic tests.

## Introduction

The determination of disease status based upon some diagnostic test is a fundamental principle in medicine. Tests may be straightforward, for example the presence or absence of crepitations on lung auscultation, or highly complex, such as the identification of specific changes in a patient’s genetic code, but very often clinicians are seeking to answer a simple question with a dichotomous answer: does this patient have or not have the disease in question?

Accordingly, very many tests have been developed which seek to provide a simple and interpretable binary result, either by identifying a target which is disease specific and the presence or absence of which confirms or refutes the diagnosis, or by providing some threshold value above (or below) which the patient can be considered positive for the disease ^1– 3^.

Binary diagnostic tests such as these rarely, if ever, perform perfectly. During development, diagnostic tests are often compared to a gold standard; a reference test or clinical diagnosis which defines true disease status for an individual. From this we can derive the fundamental performance characteristics, such as sensitivity, specificity, positive and negative predictive values and likelihood ratios. As for any other estimated quantities, principled comparison of these test metrics for two different tests requires the use of statistical inference.

Although the comparison of test metrics has been the subject of much academic inquiry, to the best of our knowledge, there is only one package for the open-source statistical programming language R ^4^ which performs this function. The DTComPair package ^5^ uses well-established statistical methods to perform statistical inference when comparing test metrics. The package is available from the Comprehensive R Archiving Network (CRAN).

A newer program, compbdt, has also been published in an open-access journal ^6^. This program uses the most up-to-date statistical methods. However, the code is presented as one large function and is therefore unavailable on CRAN. This limits the useability of the program, as users are required to search the statistical literature and be sufficiently proficient with R to import and run the function.

We sought to develop a new R package which performs both descriptive and inferential statistics for the commonly used test metrics, combining the optimised statistical methods of compbdt with the useability and availability of DTComPair.

Because the target users of this package are clinicians involved in the development and evaluation of diagnostic tests, not statisticians or computational scientists, we defined a list of features to maximise usability. Specifically, the new package is designed to:

- Take a data frame or matrix as an argument containing all commonly used binary operators (eg. yes/no, y/n, pos/neg, 1/0, etc.).
- Return output following a single function call.
- Display a contingency table (confusion matrix) summarising the raw data.
- Allow users to select whether this matrix has margins displaying row and column sums.
- Return the prevalence of the condition in question (according to the reference standard) and a confidence interval based on the cohort studied.
- Allow the user to select which pairs of test metrics they are interested in (e.g. sensitivity/specificity) and exclude those which are not relevant to their hypothesis.
- Return a matrix for each selected test metric displaying point estimates for both tests, alongside standard errors and confidence intervals.
- Return test statistics and p-values for differences between the selected test metrics for each of the two tests
- Handle multiple testing using standard correction methods.
- Offer the user the option of continuity correction if McNemar’s test is indicated.
- Allow the user to input test names to facilitate interpretation.
- Provide an optional function which interprets the output (in plain English) for the user.
- Provide an additional function for summarising descriptive statistics for one test.

Here we introduce testCompareR, a new R package which calculates the performance metrics for two diagnostic tests by comparing them against a user-provided gold standard test, then compares the performance metrics of those two binary diagnostic tests to one another, all subject to a paired experimental design.

## Methods

### Implementation

***Statistical methods***

**Calculating the test metrics**

Each of the test metrics is calculated using well-established and standardised formulas ^7– 9^, based upon standard contingency tables comparing the gold standard and the test results ( Table 1).

**Table 1.** Contingency table evaluating a test against a gold standard.

		Test	
		+	-
Gold standard	+	True positive (TP)	False negative (FN)
	-	False positive (FP)	True negative (TN)

*Diagnostic accuracies (sensitivity and specificity)*

$$Se=\frac{TP}{TP+FN}\;Sp=\frac{TN}{TN+FP}$$

*Predictive values*

$$PPV=\frac{TP}{TP+FP}\;NPV=\frac{TN}{TN+FN}$$

*Likelihood ratios*

$$PLR=\frac{Se}{1-Sp}\;NLR=\frac{1-Se}{Sp}$$

**Estimating confidence intervals**

The diagnostic accuracies and predictive values are binomial proportions, for which several methods exist to estimate confidence intervals. Yu *et al.* (2014, see 10) proposed a modification of the Wilson interval and demonstrated superior performance compared to other commonly used intervals.

The likelihood ratios are not binomial proportions, but rather ratios of two independent binomial proportions. A comprehensive review and simulation of methods to estimate confidence intervals for the ratios of binomial proportions demonstrated that an approximation to the score method had superior performance ^11^.

These methods are implemented within the testCompareR package. For detailed mathematical descriptions, see ‘Mathematical descriptions’ at the end.

**Hypothesis testing**

*Diagnostic accuracies*

Simulation studies have demonstrated that the best methods for comparing diagnostic accuracies obtained from paired data vary depending on prevalence and total number of participants ^12^.

As a rule of thumb, in cases where prevalence is low (<10%) and the total number of participants is less than 100, the Wald test should be used to test two null hypotheses ^12^:

$$H_{0}:Se_{1}=Se_{2}$$

$$H_{0}:Sp_{1}=Sp_{2}$$

Where either condition remains unmet the optimal method involves first testing the global null hypothesis:

$$H_{0}:Se_{1}=Se_{2}\;\text{and}\;Sp_{1}=Sp_{2}$$

$$H_{1}:Se_{1}\neq Se_{2}\;\text{or}\;Sp_{1}\neq Sp_{2}$$

**Figure 1.**
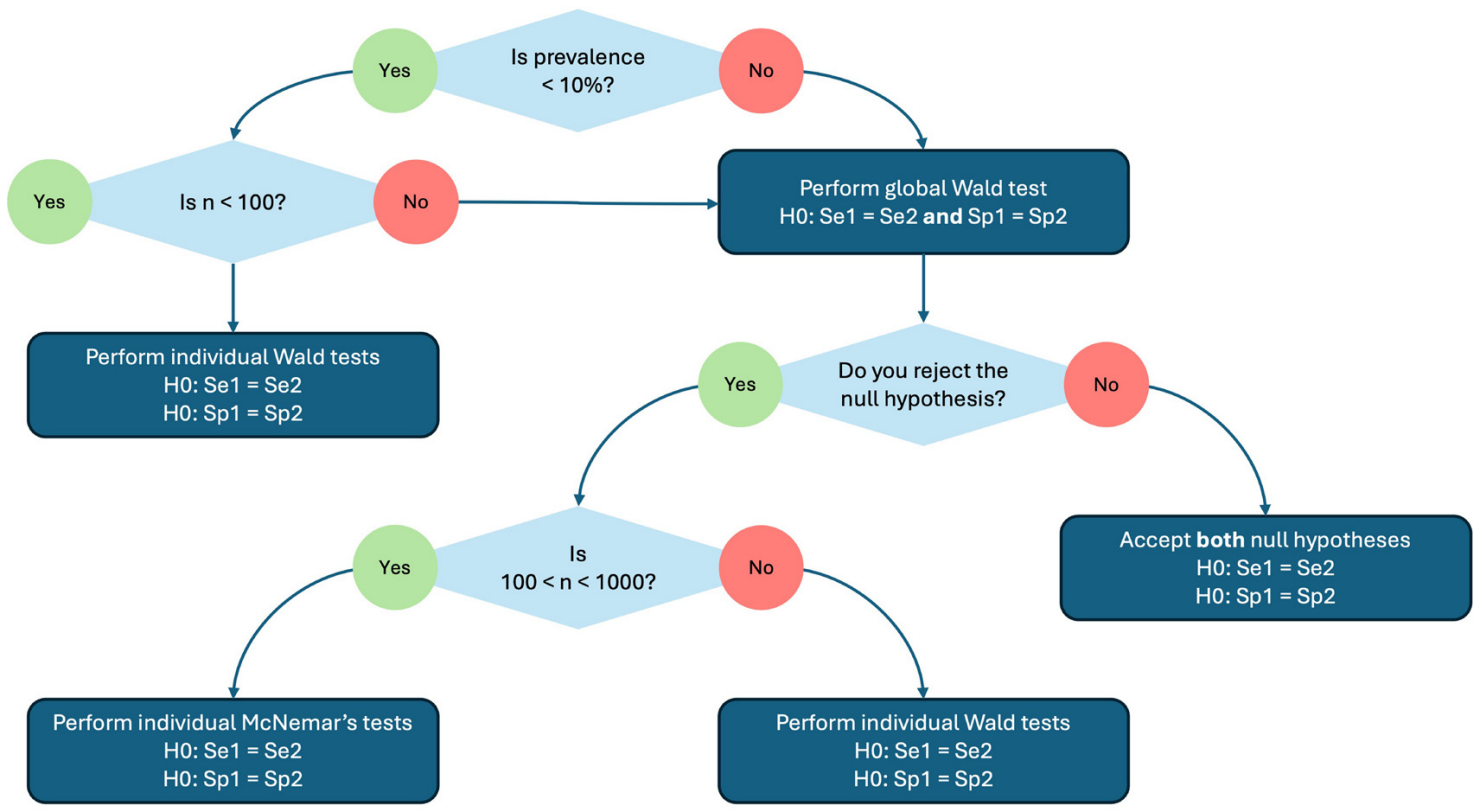
A decision tree showing how the hypothesis testing for diagnostic accuracies (sensitivity and specifity) is performed by the testCompareR package.

The Wald test statistic forms the basis of this test. If the global null hypothesis is not rejected then neither individual null hypothesis should be considered unmet. When the global null is rejected then individual hypothesis tests are performed to determine whether the sensitivities are significantly different, or the specificities or both. When the total number of participants is less than or equal to 100, or greater than or equal to 1000, then the Wald test statistic performs best according to Roldán-Nofuentes and Sidaty-Regad ^12^. In cases where total number of participants is between 100 and 1000 then McNemar’s test should be used ^12^. In the testCompareR package, McNemar’s test is performed with continuity correction by default. A decision tree summarising this process is shown in Figure 1.

*Predictive values*

In a manner similar to that seen for diagnostic accuracies, the approach to hypothesis testing for the predictive values relies upon the Wald test statistic to first perform a global hypothesis test ^13^. If the global hypothesis is rejected, the causes of significance are investigated using a weighted generalised score statistic, as described by Kosinski ^14^.

*Likelihood ratios*

The testCompareR package also uses global hypothesis testing to compare the likelihood ratios. The global hypothesis test considers the natural logarithm of the ratios of the positive likelihood ratios and negative likelihood ratios before calculating the Wald test statistic ^15^. Where the global null is rejected the cause of significance is determined by applying the same statistical methods individually.

***Installation***

testCompareR is available from CRAN and can be installed via the install.packages() function. This version should be the preferred version for most users. The development version with the most current features is available from GitHub.


# install from CRAN
install.packages("testCompareR")

# install development version
if(require("devtools")) {
  install_github("kajlinko/testCompareR")
} else {
  install.packages("devtools")
  require("devtools")
  install_github("kajlinko/testCompareR")
}


***Data preparation***

Flexible data entry is one of the key features of testCompareR. This minimises the number of pre-processing steps required by the user. In fact, for users not proficient with R, pre-processing could be handled entirely within spreadsheet or database software. There are only two steps which are imperative.

Firstly, positive and negative results must be coded according to a list of acceptable values. This list is relatively extensive, incorporating commonly used synonyms for coding positive and negative results in the English language (see Table 2). There is no requirement for consistency, which may benefit researchers performing secondary data analyses using data collated from multiple sources. Additionally, the package handles cases and white space so that researchers do not have to manually or computationally re-code their data.

**Table 2.** List of acceptable values when coding data from binary diagnostic tests. Values in inverted commas indicate character strings. Integer values are denoted without quotation marks. The acceptable values are not case sensitive, e.g. “pos”, “Pos”, “POS” would all be acceptable for positive result coding.

	List of acceptable values
Positive	"positive", "pos", "p", "yes", "y", "+", "1", "true", "t", 1
Negative	"negative", "neg", "no", "n", "-", "0", "2", "false", "f", 0, 2

Secondly, the structure of the data as presented to the package must conform to two rules:

1) The data need to be paired, i.e. the results on each row are for the same test sample or individual.
2) The observations on each individual row are independent, i.e. no biological or technical replicates, which violate the assumption of independence.

Data can either be provided as a data frame (to the df argument) or as a series of vectors (to the test1, test2 and gold arguments. Users who supply a data frame to the df argument may choose to specify the columns which should be used for analysis, by passing strings or integers to test1, test2 and gold. In the case where columns are not specified compareR will default to columns one, two and three for test1, test2 and gold, respectively, while issuing a warning to inform the user to check that this is correct. Failure to comply with rules 1 and 2 may produce sensible-looking results which do not answer the question asked by the researcher. Users should therefore take extra care to ensure their data are appropriate for this method before implementing their analysis.

testCompareR currently expects complete data to be provided to the functions. The user is required to decide how to deal with missing values (e.g. complete case analysis, single value imputation, multiple imputation). If in doubt, users of the package should discuss their individual situation with an experienced statistician.

***Mathematical descriptions***

Here we describe the mathematical equations for each of the confidence intervals calculated in the testCompareR paper.

First, we must define the contingency table which compares both tests under evaluation against the gold standard. This defines the fundamental values which will be used in subsequent calculations (see Table 3).

**Table 3.** Table of fundamental values. Each value represents the number of individuals meeting the conditions. For example, s11 represents the number of individuals for whom the gold standard, Test 1 and Test 2 are all positive.

		Test 1 +		Test 1 -		Column totals
		Test 2 +	Test 2 -	Test 2 +	Test 2 -	
Gold standard	+	s11	s10	s01	s00	ss
	-	r11	r10	r01	r00	rr
Row totals		n11	n10	n01	n00	n

**Sensitivity (Yu *et al.* interval):**

$$\;1.\;0.5+\frac{s+z_{1-\alpha /2}^{4}/53}{s+z_{1-\alpha /2}^{4}}(\hat{S}e_{i}-0.5)\pm \frac{z_{1-\alpha /2}}{s+z_{1-\alpha /2}^{2}}\sqrt{s(1-\hat{S}e_{i})\hat{S}e_{i}+\frac{z_{1-\alpha /2}^{2}}{4}}$$

where *z*_1– *α*/2_ is the 100(1 – *α*/2)th percentile of a standard normal distribution, *s* represents the total number of positive cases (ie. *s _11 _* + *s _10_* for test 1 and *s _11_* + *s _01_* for test 2) and *Se _i_* represents the point estimate of sensitivity for test 1 or test 2.

**Specificity (Yu *et al.* interval):**

$$\;2.\;0.5+\frac{r+z_{1-\alpha /2}^{4}/53}{r+z_{1-\alpha /2}^{4}}(\hat{S}p_{i}-0.5)\pm \frac{z_{1-\alpha /2}}{r+z_{1-\alpha /2}^{2}}\sqrt{r(1-\hat{S}p_{i})\hat{S}p_{i}+\frac{z_{1-\alpha /2}^{2}}{4}}$$

where *z*_1– *α*/2_ is the 100(1 – *α*/2)th percentile of a standard normal distribution, *r* represents the total number of negative cases (ie. *r _00_* + *r _01_* for test 1 and *r _00_* + *r _10_* for test 2) and *Sp _i_* represents the point estimate of specificity for test 1 or test 2.

**Positive predictive value (Yu *et al.* interval):**

$$\;3.\;0.5+\frac{n_{1\cdot }+z_{1-\alpha /2}^{4}/53}{n_{1\cdot }+z_{1-\alpha /2}^{4}}(P\hat{P}V_{1}-0.5)\pm \frac{z_{1-\alpha /2}}{n_{1\cdot }+z_{1-\alpha /2}^{2}}\sqrt{n_{1\cdot }(1-P\hat{P}V_{1})P\hat{P}V_{1}+\frac{z_{1-\alpha /2}^{2}}{4}}$$

$$\;4.\;0.5+\frac{n_{\cdot 1}+z_{1-\alpha /2}^{4}/53}{n_{\cdot 1}+z_{1-\alpha /2}^{4}}(P\hat{P}V_{2}-0.5)\pm \frac{z_{1-\alpha /2}}{n_{\cdot 1}+z_{1-\alpha /2}^{2}}\sqrt{n_{\cdot 1}(1-P\hat{P}V_{2})P\hat{P}V_{2}+\frac{z_{1-\alpha /2}^{2}}{4}}$$

where *n*_1_. = *n*_11_ + *n*_10_ and *n*. _1_ = *n*_11_ + *n*_01_.

**Negative predictive value (Yu *et al.* interval):**

$$\;5.\;0.5+\frac{n_{0\cdot }+z_{1-\alpha /2}^{4}/53}{n_{0\cdot }+z_{1-\alpha /2}^{4}}(N\hat{P}V_{1}-0.5)\pm \frac{z_{1-\alpha /2}}{n_{0\cdot }+z_{1-\alpha /2}^{2}}\sqrt{n_{0\cdot }(1-N\hat{P}V_{1})N\hat{P}V_{1}+\frac{z_{1-\alpha /2}^{2}}{4}}$$

$$\;6.\;0.5+\frac{n_{\cdot 0}+z_{1-\alpha /2}^{4}/53}{n_{\cdot 0}+z_{1-\alpha /2}^{4}}(N\hat{P}V_{2}-0.5)\pm \frac{z_{1-\alpha /2}}{n_{\cdot 0}+z_{1-\alpha /2}^{2}}\sqrt{n_{\cdot 0}(1-N\hat{P}V_{2})N\hat{P}V_{2}+\frac{z_{1-\alpha /2}^{2}}{4}}$$

where *n*_0_. = *n*_00_ + *n*_01_ and *n*. _0_ = *n*_00_ + *n*_10_.

**Positive likelihood ratio (approximation to the score method):**

$$\;7.\;\frac{\tilde{n}{\tilde{s}}_{1\cdot }{\tilde{r}}_{1\cdot }+\frac{z_{1-\alpha /2}^{2}}{2}(\tilde{s}{\tilde{s}}_{1\cdot }+\tilde{r}{\tilde{r}}_{1\cdot }\prime -2{\tilde{s}}_{1\cdot }{\tilde{r}}_{1\cdot })\pm z_{1-\alpha /2}\sqrt{{\tilde{n}}^{2}{\tilde{s}}_{1\cdot }{\tilde{r}}_{1\cdot }\left[{\tilde{s}}_{1\cdot }+{\tilde{r}}_{1\cdot }-\tilde{n}\tilde{S}e_{1}(1-\tilde{S}p_{1})\right]+\frac{z_{1-\alpha /2}^{2}}{4}{\left(\tilde{s}{\tilde{s}}_{1\cdot }-\tilde{r}{\tilde{r}}_{1\cdot }\right)}^{2}}}{{\tilde{r}}_{1\cdot }\left[\tilde{n}\tilde{s}(1-\tilde{S}p_{1})-z_{1-\alpha /2}^{2}(\tilde{s}-{\tilde{r}}_{1\cdot })\right]}$$

where $\tilde{s}$ _1_. = *s*_1_. + 0.5, $\tilde{r}$ _1_. = *r*_1_. + 0.5, $\tilde{s}$ = *s* + 1, $\tilde{r}$ = *r* + 1, $\tilde{n}$ = *n* + 2, $\tilde{S}$ *e*_1_ = $\tilde{s}$ _1_./ $\tilde{s}$ and $\tilde{S}$ *p*_1_ = $\tilde{r}$ _0_./ $\tilde{r}$ .

Regarding *PLR*_1_, if the lower limit of the confidence interval is less than $\tilde{s}$ _1_./( $\tilde{n}$ – $\tilde{r}$ _1_.) or greater than ${\hat{PLR}}_{1}$ then the lower limit is given by:

$$\;8.\;\frac{{\tilde{s}}_{1\cdot }(1-\tilde{S}p_{1})+\frac{z_{1-\alpha /2}^{2}}{2}-z_{1-\alpha /2}\sqrt{\frac{z_{1-\alpha /2}^{2}}{4}+{\tilde{s}}_{1\cdot }(1-\tilde{S}p_{1}-\tilde{S}e_{2})}}{\tilde{S}{\left(1-\tilde{S}p_{1}\right)}^{2}+z_{1-\alpha /2}^{2}}$$

Equally, if the upper limit is greater than ( $\tilde{n}$ – $\tilde{s}$ _1_.)/ $\tilde{r}$ _1_. or less than ${\hat{PLR}}_{1}$ then the upper limit is given by:

$$\;9.\;\frac{{\tilde{r}}_{1\cdot }\tilde{S}e_{1}+\frac{z_{1-\alpha /2}^{2}}{2}+z_{1-\alpha /2}\sqrt{\frac{z_{1-\alpha /2}^{2}}{4}+{\tilde{r}}_{1\cdot }(\tilde{S}e_{1}+\tilde{S}p_{1}-1)}}{\tilde{r}{\left(1-\tilde{S}p_{1}\right)}^{2}}$$

Replacing *x*_1_. with *x*. _1_ where *x* represents any of the fundamental values *s*, *r* or *n* and $\tilde{S}$ *e*_1_ and $\tilde{S}$ *p*_1_ with $\tilde{S}$ *e*_2_ and $\tilde{S}$ *p*_2_, respectively, will return the values for ${\hat{PLR}}_{2}$ .

**Negative likelihood ratio (approximation to the score method):**

$$\;10.\;\frac{\tilde{n}{\tilde{s}}_{0\cdot }{\tilde{r}}_{0\cdot }+\frac{z_{1-\alpha /2}^{2}}{2}(\tilde{s}{\tilde{s}}_{0\cdot }+\tilde{r}{\tilde{r}}_{0\cdot }-2{\tilde{s}}_{0\cdot }{\tilde{r}}_{0\cdot })\pm z_{1-\alpha /2}\sqrt{{\tilde{n}}^{2}{\tilde{s}}_{0\cdot }{\tilde{r}}_{0\cdot }\left[{\tilde{s}}_{0\cdot }+{\tilde{r}}_{0\cdot }-\tilde{n}(1-\tilde{S}e_{1})\tilde{S}p_{1}\right]+\frac{z_{1-\alpha /2}^{2}}{4}{\left(\tilde{s}{\tilde{s}}_{0\cdot }-\tilde{r}{\tilde{r}}_{0\cdot }\right)}^{2}}}{{\tilde{r}}_{0\cdot }\left[\tilde{n}\tilde{s}\tilde{S}p_{1}-z_{1-\alpha /2}^{2}(\tilde{s}-{\tilde{r}}_{0\cdot })\right]}$$

where $\tilde{s}$ _0_. = *s*_0_. + 0.5, $\tilde{r}$ _0_. = *r*_0_. + 0.5, $\tilde{s}$ = *s* + 1, $\tilde{r}$ = *r* + 1, $\tilde{n}$ = *n* + 2, $\tilde{S}$ *e*_1_ = $\tilde{s}$ _1_./ $\tilde{s}$ and $\tilde{s}$ *p*_1_ = $\tilde{r}$ _0_./ $\tilde{r}$ .

Regarding *NLR*_1_, if the lower limit of the confidence interval is less than $\tilde{s}$ _0_./( $\tilde{n}$ – $\tilde{r}$ _0_.) or greater than $N\hat{L}R_{1}$ then the lower limit is given by:

$$\;11.\;\frac{{\tilde{s}}_{0\cdot }\tilde{S}p_{1}+\frac{z_{1-\alpha /2}^{2}}{2}-z_{1-\alpha /2}\sqrt{\frac{z_{1-\alpha /2}^{2}}{4}+{\tilde{s}}_{0\cdot }(\tilde{S}p_{1}+\tilde{S}e_{1}-1)}}{\tilde{s}\tilde{S}p_{1}^{2}+z_{1-\alpha /2}^{2}},$$

Equally, if the upper limit is greater than ( $\tilde{n}$ – $\tilde{s}$ _0._)/ $\tilde{r}$ _0_. or less than ${\hat{PLR}}_{2}$ then the upper limit is given by:

$$\;12.\;\frac{{\tilde{r}}_{0\cdot }(1-\tilde{S}e_{1})+\frac{z_{1-\alpha /2}^{2}}{2}+z_{1-\alpha /2}\sqrt{\frac{z_{1-\alpha /2}^{2}}{4}+{\tilde{r}}_{0\cdot }(1-\tilde{S}e_{1}-\tilde{S}p_{1})}}{\tilde{r}\tilde{S}p_{1}^{2}}.$$

Replacing *x*_0_. with *x*. _0_ where *x* represents any of the fundamental values *s*, *r* or *n* and $\tilde{S}$ *e*_1_ and $\tilde{S}$ *p*_1_ with $\tilde{S}$ *e*_2_ and $\tilde{S}$ *p*_2_, respectively, will return the values for $N\hat{L}R_{2}$ .

***Using the package***

The package consists of three main functions.

compareR(): This is the workhorse function of the package. It takes as its argument a data frame or matrix, which should be appropriately formatted (see ‘Data preparation’). Internal functions then ensure data are correctly coded, before calculating output values according to the methodologies previously described. A range of optional parameters allow users to customise the output:

- df A data frame or matrix with at least 3 columns. Can be subset using test1, test2 and gold arguments. If test1, test2 and gold remain NULL then defaults to test1 = df[,1], test2 = df[,2] and gold = df[,3]. Flexible coding of positive and negative results permitted.
- test1 Either a vector of values for Test 1 (if df is NULL) or a string or integer value to be used for subsetting df.
- test2 Either a vector of values for Test 2 (if df is NULL) or a string or integer value to be used for subsetting df.
- gold Either a vector of values for the gold standard test (if df is NULL) or a string or integer value to be used for subsetting df.
- alpha An alpha value, i.e. significance level. Defaults to 0.05.
- margins A Boolean value indicating whether the contingency tables should have margins containing summed totals of rows and columns. Defaults to FALSE.
- multi_corr Method for multiple comparisons. Uses p.adjust.methods. Defaults to “holm”.
- cc A Boolean value indicating whether McNemar’s test should be applied with continuity correction. Defaults to TRUE.
- dp Number of decimal places of output in summary tables. Defaults to 1.
- sesp A Boolean value indicating whether output should include sensitivity and specificity. Defaults to TRUE.
- ppvnpv A Boolean value indicating whether output should include positive and negative predictive values. Defaults to TRUE.
- plrnlr A Boolean value indicating whether output should include positive and negative likelihood ratios. Defaults to TRUE.
- conf.int A character string, either “contemporary” or “classic”. Indicates whether the function should use the statistical methods described herein (contemporary, see Mathematical methods) or the exact binomial (classic) to calculate the confidence intervals.
- test.names A character vector of length two giving the names of the two different binary diagnostic tests. Defaults to c(“Test 1”, “Test 2”).

**Figure 2.**
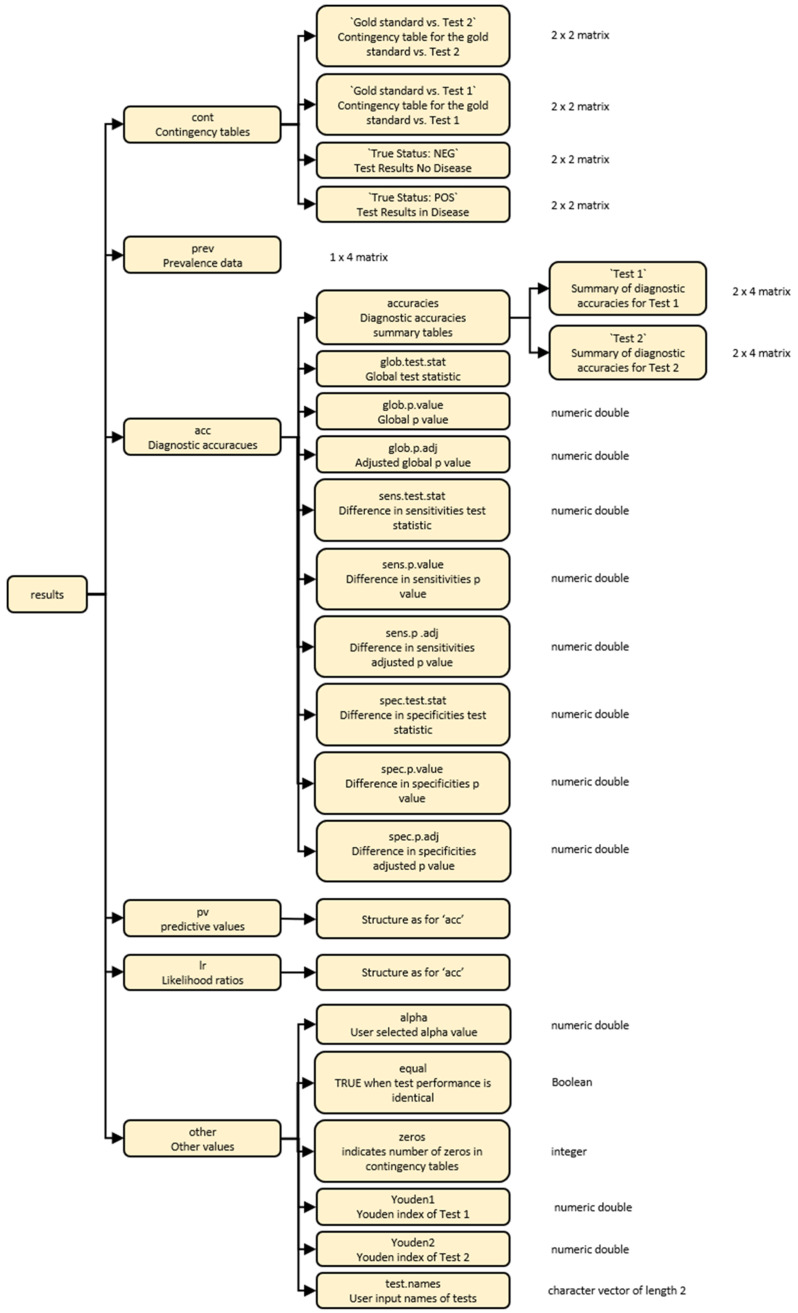
A diagrammatic representation of the multilevel list output from the compareR() function.

The output from the compareR() function is a multilevel list object of class compareR. Users can access individual results using standard R indexing. The list structure is visually described in Figure 2.

interpretR(): The interpretR() function provides a means for clinicians to quickly understand the significance of their results, without having to manually dissect the multilevel list output from compareR(). By passing the interpretR() function the output from compareR() the user is provided with a readout in the console in plain English.

summariseR(): When a clinician is evaluating only one test, the summariseR() function will quickly calculate and display the descriptive statistics. Although this is not difficult to perform manually, the summariseR() function is fast and convenient, even with large datasets. Like compareR(), summariseR() allows flexible input, which can prevent researchers having to manually re-code their data. Users should note that unlike compareR(), summariseR() requires a data frame or matrix with two columns, evaluated test and gold standard test, as input.

Because the outputs for both compareR and interpretR are verbose, testCompareR takes advantage of S3 methods within R’s object-oriented programming system to provide the most useful results in more concise outputs. Users can get a summary() of the output, print() the most pertinent results as a table or plot() a simple visualisation of the diagnostic accuracies and predictive values.

### Operation

At the time of writing, the testCompareR package can be run on any operating system that supports R version 4.3.0 or later.

## Use cases

To demonstrate the use of the testCompareR package we will use the Coronary Artery Surgery Study ( cass) ^16^ data set which is included with the package.

First, examining the data we see that the data frame contains three columns, exercise relating to an exercise stress test, cp relating to a history of chest pain (each used here as tests for coronary artery disease), and angio, which reports the outcome of the gold standard test – coronary angiography. Here, we can see that the data are already coded as zeros and ones.


data
(cass)
rbind(head(cass), tail(cass))
   exercise cp angio
1          1  1     1
2          1  1     1
3          1  1     1
4          1  1     1
5          1  1     1
6          1  1     1
866        0  0     0
867        0  0     0
868        0  0     0
869        0  0     0
870        0  0     0
871        0  0     0


To compare the two tests, pass the data to the compareR() function. This returns a multilevel list, as previously described. To avoid an unnecessary lengthy output in the example we have set the parameters ppvnpv and plrnlr to FALSE which allows us not to execute these tests.


results <- compareR(cass, ppvnpv = FALSE, plrnlr = FALSE)
results

$cont
$cont$`True Status: POS`
          Test 2
Test 1     Positive Negative
  Positive      473       29
  Negative       81       25
 
$cont$`True Status: NEG`
          Test 2
Test 1     Positive Negative
  Positive       22       46
  Negative       44      151
 
$prev
           Estimate  SE Lower CI Upper CI
Prevalence     69.8 1.6     66.7     72.8

$acc
$acc$accuracies
$acc$accuracies$`Test 1`
            Estimate  SE Lower CI Upper CI
Sensitivity     82.6 1.5     79.4     85.4
Specificity     74.1 2.7     68.6     79.1

$acc$accuracies$`Test 2`
            Estimate  SE Lower CI Upper CI
Sensitivity     91.1 1.2     88.6     93.1
Specificity     74.9 2.7     69.4     79.8

$acc$glob.test.stat
[1] 25.662

$acc$glob.p.value
[1] 2.676497e-06

$acc$glob.p.adj
[1] 2.676497e-06

$acc$sens.test.stat
[1] 23.64545

$acc$sens.p.value
[1] 0

$acc$sens.p.adj
[1] 0

$acc$spec.test.stat
[1] 0.01111111

$acc$spec.p.value
[1] 0.9911348

$acc$spec.p.adj
[1] 1

$other
$other$alpha
[1] 0.05

$other$equal
[1] FALSE

$other$zeros
[1] 0

$other$Youden1
[1] 0.5671028

$other$Youden2
[1] 0.6602336

$other$test.names
[1] "Test 1" "Test 2"

attr(,"class")
[1] "compareR"


Values in this list can be accessed via standard indexing.


results$acc$accuracies # returns matrices summarising diagnostic accuracies
$`Test 1`
            Estimate  SE Lower CI Upper CI
Sensitivity     82.6 1.5     79.4     85.4
Specificity     74.1 2.7     68.6     79.1

$`Test 2`

            Estimate SE Lower CI Upper CI
Sensitivity    91.1 1.2     88.6     93.1
Specificity    74.9 2.7     69.4     79.8


Finally, if the user prefers to see an interpretation of the output in plain English, including highlighted values where results are significant, they can pass the output of compareR() to interpretR().


interpretR(results)
--------------------------------------------------------------------------------
CONTINGENCY TABLES
--------------------------------------------------------------------------------

True Status - POSITIVE
          Test 2
Test 1     Positive Negative
  Positive      473       29
  Negative       81       25
 
True Status - NEGATIVE
          Test 2
Test 1     Positive Negative
  Positive       22       46
  Negative       44      151
 
--------------------------------------------------------------------------------
PREVALENCE (%)
--------------------------------------------------------------------------------

           Estimate  SE Lower CI Upper CI
Prevalence     69.8 1.6     66.7     72.8

--------------------------------------------------------------------------------
DIAGNOSTIC ACCURACIES
--------------------------------------------------------------------------------

 Test 1 (%)
            Estimate  SE Lower CI Upper CI
Sensitivity     82.6 1.5     79.4     85.4
Specificity     74.1 2.7     68.6     79.1

 Test 2 (%)
 
            Estimate  SE Lower CI Upper CI
Sensitivity     91.1 1.2     88.6     93.1
Specificity     74.9 2.7     69.4     79.8

Global Null Hypothesis: Se1 = Se2 & Sp1 = Sp2
Test statistic:  25.662  Adjusted p value:  2.676497e-06 ***SIGNIFICANT***

Investigating cause(s) of significance

Null Hypothesis 1: Se1 = Se2
Test statistic:  23.64545  Adjusted p value:  0 ***SIGNIFICANT***

Null Hypothesis 2: Sp1 = Sp2
Test statistic:  0.01111111  Adjusted p value:  1


testCompareR is elegant in its simplicity. Several parameters permit customisation of the output, but they are not elaborated here as they are not essential to understand the workings of the package (the interested reader is referred to the testCompareR package documentation files). Further details can be found within the package vignette, which contains examples for all modifiable parameters.

## Results

### Evaluation

We calculated the results for the Coronary Artery Surgery Study ( cass) dataset using testCompareR, DTComPair and compbdt. This dataset looks at exercise stress testing and history of chest pain as two tests for coronary artery disease as determined by coronary angiography (the gold standard) ^16^. It has become a standard for testing in statistical research regarding test metrics. The results are shown in Table 4.

**Table 4.** Outputted values for each of the test metrics by package/program. DTComPair we were unable to retrieve prevalence from DTComPair’s standard functions. For likelihood ratios testCompareR and compbdt report SE, whereas DTComPair reports SE.log, therefore they are not directly comparable. Se - sensitivity, SE - standard error, LCI - lower confidence interval, UCI - upper confidence interval, p - p value, Sp - specificity, PPV - positive predictive value, NPV - negative predictive value, PLR - positive likelihood ratio, NLR - negative likelihood ratio.

Disease prevalence										
	Prevalence	SE	LCI	UCI						
testCompareR	69.8	1.6	66.7	72.8						
compbdt	69.8	1.6	66.7	72.8						
Diagnostic accuracies: Test 1										
	Se	SE	LCI	UCI	p	Sp	SE	LCI	UCI	p
testCompareR	82.6	1.5	79.4	85.4	<0.0001	74.1	2.7	68.6	79.1	0.92
DTComPair	82.6	1.5	79.6	85.6	<0.0001	74.1	2.7	68.9	79.4	0.83
compbdt	82.6	1.5	79.4	85.4	<0.0001	74.1	2.7	68.6	79.1	0.99
Diagnostic accuracies: Test 2										
	Se	SE	LCI	UCI	p	Sp	SE	LCI	UCI	p
testCompareR	91.1	1.2	88.6	93.2		74.9	2.7	69.4	79.8	
DTComPair	91.1	1.2	88.9	93.2		74.9	2.7	69.7	80.1	
compbdt	91.1	1.2	88.6	93.2		74.9	2.7	69.4	79.8	
Predictive values: Test 1										
	PPV	SE	LCI	UCI	p	NPV	SE	LCI	UCI	p
testCompareR	88.1	1.4	85.2	90.5	0.37	64.8	2.8	59.3	70.0	<0.0001
DTComPair	88.1	1.4	85.4	90.7	0.37	64.8	2.8	59.4	70.2	<0.0001
compbdt	88.1	1.4	85.2	90.5	0.37	64.8	2.8	59.3	70.0	<0.0001
Predictive values: Test 2										
	PPV	SE	LCI	UCI	p	NPV	SE	LCI	UCI	p
testCompareR	89.4	1.2	86.7	91.6		78.5	2.6	73.0	83.2	
DTComPair	89.4	1.2	86.9	91.8		78.5	2.6	73.4	83.6	
compbdt	89.4	1.2	86.7	91.6		78.5	2.6	73.0	83.2	
Likelihood ratios: Test 1										
	PLR		LCI	UCI	p	NLR		LCI	UCI	p
testCompareR	3.2		2.6	4.0	0.37	0.23		0.20	0.28	<0.0001
DTComPair	3.2		2.6	3.9	0.37	0.23		0.20	0.28	<0.0001
compbdt	3.2		2.6	4.0	0.37	0.23		0.20	0.28	<0.0001
Likelihood ratios: Test 2										
	PLR		LCI	UCI	p	NLR		LCI	UCI	p
testCompareR	3.6		3.0	4.5		0.12		0.09	0.15	
DTComPair	3.6		2.9	4.5		0.12		0.09	0.15	
compbdt	3.6		3.0	4.5		0.12		0.09	0.15	

Both testCompareR and DTComPair both achieve similar results. Given that the mathematical basis of testCompareR and compbdt is the same, we see almost identical results. However, the test statistics for the individual hypothesis tests of the diagnostic accuracies (sensitivity and specificity) are distributed approximately according to the chi-squared distribution, which is reflected in the testCompareR package. The original compbdt program mistakenly treats these test statistics as if they were distributed according to the normal distribution which can lead to differences between results (see e.g. results for the diagnostic accuracy of Test 1 in Table 4).

### Performance evaluation

**Figure 3.**
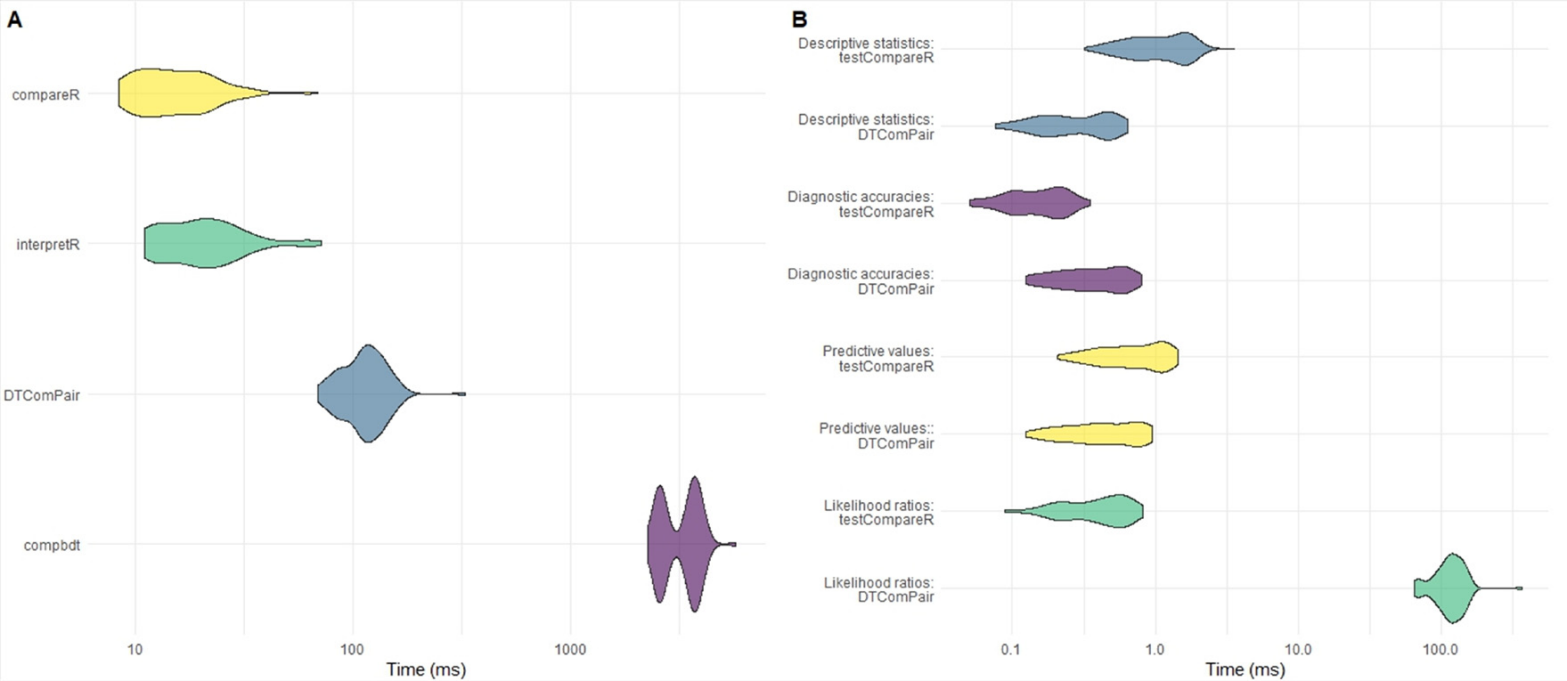
**A**) Comparison of computational time for testCompareR, DTComPair and compbdt. Each package was run 100 times. **B**) Comparison of testCompareR and DTComPair, by function. testCompareR compares the likelihood ratios more quickly than DTComPair because it uses a method based on an approximation of the score statistic, which requires only solving of a second degree equation. The DTComPair method is based upon logistical regression. Each function was run 100 times.

To evaluate the performance of the package we used the microbenchmark package ^17^ to repeatedly compute the test metrics, calculate confidence intervals and perform inferential tests on the cass dataset using testCompareR, DTComPair and compbdt. Each set of calculations was repeated 100 times and the time elapsed for each test was recorded. The results are shown in Table 5 and Figure 3A.

**Table 5.** Time to compute descriptive and inferential statistics. Times computed using the cass dataset for individual packages/programs using a Windows 10 x64 laptop with 16GB RAM and an 11th Gen Intel(R) Core(TM) i7-1165G7 @ 2.80GHz processor. testCompareR was run in R 4.3.0 via Rstudio 2023.12.1+402.

	Mean (ms)	SD (ms)
compareR	14.1	7.8
interpretR	20.5	11.4
DTComPair	114.2	38.1
compbdt	3,578.4	1,752.3

A certain amount of pre-processing was required in order that the data conformed to the requirements of each package or program. The code describing this pre-processing is included with this paper.

Our results demonstrate that the compareR() function returns the results considerably quicker than either DTComPair or compbdt. This performance advantage is maintained even when compareR() is wrapped by the interpretR() function. Further testing of the individual functions from DTComPair and the internal functions of testCompareR demonstrated that the cause of the difference between the two packages is the method for comparing the likelihood ratios, shown in Figure 3B. The method used by DTComPair is based on logistical regression, whereas the method used by testCompareR is based on an approximation of the score statistic, which is simpler to compute requiring only solving of a second-degree equation. Simulation to estimate the power and type 1 error rate for this approximation across a large range of scenarios found that it performs well in most reasonable use cases ^15^.

## Discussion

Despite the common use of binary diagnostic tests in medicine only one package, DTComPair, and one open-source program, compbdt, provide methods to compare the test metrics between two binary diagnostic tests using paired data ^5, 6^. This package requires the user to be computationally literate, as several function calls are necessary to extract the outputs that would normally be published when comparing the performance of two tests. Specifically, the data must be pre-processed adequately and fed to the package as an object of class tab.paired, which is specific to the package. It would be more intuitive if DTComPair accepted as its input a more general input data class, such as a data frame, matrix or a tibble. Additionally, though the package implements well-established traditional methods, the evidence suggests that newer methods provide better coverage in the case of confidence intervals and better asymptotic performance in the case of hypothesis tests ^10– 15^. Here we have shown with an example dataset that the newer methods for comparing the likelihood ratios between two tests achieve comparable results, but are more computationally efficient.

The compbdt program requires the user to find the program in the statistical literature, copy or download the code, load the function, preprocess the data and then run the function. Customisations to the output require the user to update the code. The output is in the form of a lengthy console readout, which can make downstream analyses challenging. By re-structuring the internal mechanisms, we have increased computational speed while providing additional features: testCompareR can be installed directly from CRAN; accepts a dataframe as an argument; requires minimal preprocessing; and users can customise the output through a range of well-documented optional arguments. The user can choose whether to receive their output in list form, allowing them to access individual elements for downstream analysis via indexing, or as a plain English summary, facilitating rapid interpretation of the results.

testCompareR adds to the arsenal of tools for researchers who wish to rapidly develop and evaluate diagnostic tests. By minimising the number of steps required for analysis, testCompareR frees up valuable time for laboratory and clinical research.

## Ethics and consent

This research involved neither human nor animal participants so no consent or ethical approvals were required.

## Data Availability

The data described in this paper is available within the package. The data was originally presented by Weiner *et al.* as part of the Coronary Artery Surgery Study (CASS) ^16^. This study used data from a national registry set-up and maintained by the Division of Heart & Vascular Disease at the Heart, Lung and Blood Institute of the National Institute of Health (USA). Source code available from: https://www.github.com/Kajlinko/testCompareR Archived software available from: https://zenodo.org/doi/10.5281/zenodo.11488420 ^18^. License: GPL-3.0+
