# Supplementary material for: testCompareR: an R package to compare two binary diagnostic tests using paired data
Source: Wellcome Open Res. 2024 Nov 26;9:351. Originally published 2024 Jul 2. [Version 4] doi: 10.12688/wellcomeopenres.22411.4 (PMC11535492; doi:10.12688/wellcomeopenres.22411.4)
Supplement: Peer review history

# Reviewer report: Reviewer response for version 4

**DOI:** 10.21956/wellcomeopenres.25879.r115312

**Published:** 2024-12-30

**Authors:** Abel Torres Espín

**Affiliations:** 1 University of Waterloo, Waterloo, ON, Canada

The article presents the testCompareR R package developed by the authors to provide an accessible implementation of modern hypothesis testing for the comparison of two binary tests referenced to a gold standard. The package is intuitive, easy to use, and provides nice features such as flexibility in coding input values and printing organized test summaries for easy interpretation. In addition, the main functions in the package are computationally more efficient than other available implementations. Overall, the package adds a useful tool to the R programming environment. The article is well-written, and it has been reviewed and updated a few times already. I have a few comments:

**Abstract:**

- If I understood, *compbdt* implements the same mathematical approach. If that is correct, the sentence in the background “…have not yet been implemented in a statistical package.” can be misleading to some readers. I understand *combat is not an R package *but rather a function; however, that distinction might not be obvious to some readers. I suggest changing the sentence to something along the lines of: “… although software implementation exists, it has not yet been implemented as an easy-to-use R package.
- Authors might want to comment on the results of comparing testCompareR with compbdt. For example, mentioning that results are the same because uses the same mathematical approach, but computationally is ~255 times more efficient, while easier to install and to use.

**Introduction:**

- There seems to be a typo on page 5 that reads “Presence of absence…”. I believe it should read “Presence or absence…”.
- It would be clearer for the reader if Table 1 is placed in the introduction before methods.

**Methods:**

- It would be clearer for the reader if Table 2 is placed right after it is referenced before “Diagnostic accuracies…” so that the metrics from contingency tables are explained after the Table is shown.
- Table 3/data preparation section. At first, I was confused about the choice of 2 = negative as one of the options, as I have often encountered coding the other way around (1 = negative/false, 2 = positive/true). For example, a factor vector in r would codify negative/false as 1 by default, as levels are ordered alphabetically. However, it is quickly evident that having 1 = negative would interfere with the 1 = positive code in the case of 0 = negative and 1 = positive. To avoid confusion by the user and provide even more flexibility, the authors could consider incorporating an argument allowing the user to declare the list of values to use if there are values that do not follow the current list of acceptable values. That allows the user to consider any coding they please without the need to transform data in a scenario that does not follow the proposed list of values.
- I suggest changing the reference from “clinicians” to “users” through the text. Although I understand the authors' intentions, there is no reason to target clinicians as sole potential package users. For example, data scientists, epidemiologists, or biostatisticians developing classification models could also be users.

Authors mention options to use generic methods *summary(), print(),* and *plot() *for the object of class compareR. It would be good for the reader to have examples of the outputs of such method functions, especially for the plot, as a visualization of the diagnostic accuracies and predictive values are mentioned but not shown. I cannot find examples of those are in the vignettes either.

Are the conclusions about the tool and its performance adequately supported by the findings presented in the article?

Yes

Is the rationale for developing the new software tool clearly explained?

Yes

Is the description of the software tool technically sound?

Yes

Are sufficient details of the code, methods and analysis (if applicable) provided to allow replication of the software development and its use by others?

Yes

Is sufficient information provided to allow interpretation of the expected output datasets and any results generated using the tool?

Yes

Reviewer Expertise:

Health data science and public health

I confirm that I have read this submission and believe that I have an appropriate level of expertise to confirm that it is of an acceptable scientific standard.

# Reviewer report: Reviewer response for version 4

**DOI:** 10.21956/wellcomeopenres.25879.r113424

**Published:** 2024-12-13

**Authors:** Md S. Warasi

**Affiliations:** 1 Radford University, Radford, USA

The article presents an R package that can be used to calculate the sensitivity, specificity, and other operating characteristics of diagnostic assays. To compare the accuracies of two assays, the functions provided in the package can compute inferential statistics, such as p-value and confidence intervals. The package has been compared with two similar packages in terms of the assay accuracy results and computing time. While the work may not be highly innovative, it offers considerable contribution and has the potential to be useful in practical applications.

 I have a few comments, which are mainly related to clarifications and editorial improvements:

 a). Does the package assume that the “gold standard” assay, which is used as a reference assay, is perfect (i.e., free of false negatives/positives)? If so, the authors should clearly state this and consider adding it to the list of requirements at the beginning of page 8 or somewhere else. Also, the authors should discuss the implications if this assumption is violated.

 b). On page 6, the authors say “…. Wald test should be used to test two null hypotheses: …” Are these two tests performed independently in the package? Please clarify.

 c). The notation on pages 8-9 could be substantially improved if it were written using LaTeX. If possible, consider rewriting the notation section using LaTeX.

 d). When the hat and tilde symbols are used, they should be on the entire notation. However, subscripts and superscripts should be placed outside the hat and tilde symbols. Here are some examples of correct usage of such notation:

,
,
,

e). Is it necessary to describe the function arguments in the paper (page 10)? If these arguments are already explained in the package’s documentation or vignette, repeating them in the paper may be redundant and can be removed.

 f). On page 10, the ending parenthesis is missing: “…. or as a series of vectors (to the test1, test2 and gold arguments.”

 g). On page 4 (2 ^nd^ paragraph), did you mean to write “positive (or negative)” corresponding to “threshold value above (or below)”?

 h). On page 5, did you mean to write “Presence or absence” rather than “Presence of absence”?

 i). The package could be easily transformed into a software application using the shiny package in R, which would make it more accessible to clinicians. The authors might consider adding this discussion as a potential future direction in the Discussion section.

Are the conclusions about the tool and its performance adequately supported by the findings presented in the article?

Yes

Is the rationale for developing the new software tool clearly explained?

Yes

Is the description of the software tool technically sound?

Yes

Are sufficient details of the code, methods and analysis (if applicable) provided to allow replication of the software development and its use by others?

Yes

Is sufficient information provided to allow interpretation of the expected output datasets and any results generated using the tool?

Yes

Reviewer Expertise:

Probability and Statistics

I confirm that I have read this submission and believe that I have an appropriate level of expertise to confirm that it is of an acceptable scientific standard.

# Reviewer report: Reviewer response for version 4

**DOI:** 10.21956/wellcomeopenres.25879.r113349

**Published:** 2024-11-29

**Authors:** Haydar Demirhan

**Affiliations:** 1 RMIT University, Melbourne, Victoria, Australia

The authors have sufficiently addressed my points on Version 3. Thanks.

Are the conclusions about the tool and its performance adequately supported by the findings presented in the article?

Partly

Is the rationale for developing the new software tool clearly explained?

Partly

Is the description of the software tool technically sound?

Yes

Are sufficient details of the code, methods and analysis (if applicable) provided to allow replication of the software development and its use by others?

Yes

Is sufficient information provided to allow interpretation of the expected output datasets and any results generated using the tool?

Partly

Reviewer Expertise:

Statistical software, Bayesian modelling, bioinformatics, categorical data analysis.

I confirm that I have read this submission and believe that I have an appropriate level of expertise to confirm that it is of an acceptable scientific standard.

# Reviewer report: Reviewer response for version 3

**DOI:** 10.21956/wellcomeopenres.25667.r105033

**Published:** 2024-11-04

**Authors:** Haydar Demirhan

**Affiliations:** 1 RMIT University, Melbourne, Victoria, Australia

This study introduces an R package called testCompareR to compare the test metrics using two binary diagnostic tests. The features of the packages are discussed, and the statistical methods behind the proposed package are given clearly. Some examples are given to demonstrate the usage of the main functions of the package. My comments and questions are as follows:

 1. The meaning of "robust statistical package" is unclear in the abstract.

 2. In the abstract - background, it is mentioned that "...performing inferential statistics to compare commonly used test metrics for two diagnostic tests have not yet been implemented in a robust statistical package." However, in the abstract - methods, it is mentioned that another alternative is available. 

 3. The features of testCompareR are listed in the introduction. However, how many of these features are also covered in DTComPair is unclear. A comparison table could be given to make the contribution of testCompareR clearer. In its current form, it is hard to follow the contribution and necessity of the proposed package.

 4. The contributions of this study to the existing literature need to be articulated clearly in the introduction.

 5. I guess the information given under the title "Mathematical descriptions" is more of "Statistical descriptions."

 6. It is hard to read Figure 2. The authors may consider changing the orientation of the figure to landscape.

 7. Does the function interpretR() have any arguments? From the help files of the package, it seems that there are no arguments. Thus, why not include another Boolean argument in compareR() to get the results interpreted?

 8. What is the reason for giving the run time of compareR() and interpret() separately in Table 5?

 9. How does the run time change when the size of the dataset is increased?

 10. What factors impact the computational efficiency of the benchmarked packages?

Are the conclusions about the tool and its performance adequately supported by the findings presented in the article?

Partly

Is the rationale for developing the new software tool clearly explained?

Partly

Is the description of the software tool technically sound?

Yes

Are sufficient details of the code, methods and analysis (if applicable) provided to allow replication of the software development and its use by others?

Yes

Is sufficient information provided to allow interpretation of the expected output datasets and any results generated using the tool?

Partly

Reviewer Expertise:

Statistical software, Bayesian modelling, bioinformatics, categorical data analysis.

I confirm that I have read this submission and believe that I have an appropriate level of expertise to confirm that it is of an acceptable scientific standard, however I have significant reservations, as outlined above.

# Response

**Published:** 2024-11-20

**Authors:** Kyle Wilson

**Affiliations:** Department of Eye & Vision Science, University of Liverpool, Liverpool, England, UK

Thank you for the detailed feedback on our manuscript. It is clear from your report that you have taken the time to review not only the paper, but the code and package documentation. The effort is greatly appreciated; your suggestions have been integrated and the package is better for it. Beneath is a point-by-point response to your comments:   

1. The statistical innovations that have been implemented in this package have previously been implemented, but in a very long function. This function is not robust because it does not permit give the user control of the output, nor does it fail elegantly. Nevertheless, the point is taken that the meaning of robust is not elaborated and it has therefore been removed to avoid ambiguity. It is equally true that these novel methods have never been implemented in an R package.
2. This sentence specifically refers to the implementation of recent statistical advances. The alternative package, DTComPair, uses more traditional methods (eg. applying McNemar’s in all cases).
3. The major innovation is the implementation of confidence intervals with better coverage and inferential statistics with superior performance in simulation studies. Including a tabular summary of features, as you suggest, does help to clarify further advantages. Thank you.
4. Hopefully, you agree that this has been addressed by the inclusion of the table you suggested in Point 3. Further clarification on the contributions is provided in the Discussion.
5. This has been changed as you suggest.
6. Figure 2 is a busy figure. After spending some time trying to orientate it in landscape, the outcomes were not satisfying. Your point remains very valid, though, so I have supplemented the caption to include instructions for extracting the list structure from R itself.
7. This simple adjustment further improves the useability of our package. Thanks.
8. Both compareR and interpretR are included in the table to demonstrate that interpreting the outcome (by passing the result to interpretR) does not significantly affect run time.
9. compareR and interpretR scale linearly. DTComPair also scales approximately linearly, but the differences in speed become much clearer in large datasets. This has been added to the text, along with a figure. The code required to produce this figure has been added to the benchmarking directory of the development version on Github. This has been updated and archived on Zenodo.
10. This has now been clarified in the text. compareR and interpretR perform quickly and changes are negligible. DTComPair is influenced by the size of the dataset, though this is likely only to matter if including analysis of the likelihood ratios.

# Reviewer report: Reviewer response for version 3

**DOI:** 10.21956/wellcomeopenres.25667.r106828

**Published:** 2024-10-23

**Authors:** Dennis Robert

**Affiliations:** 1 Director of Clinical Research (AI), Qure.ai, Bangalore, India

Thank you for incorporating my suggestions. The revised version of the article is much improved version. I have no further comments.

Are the conclusions about the tool and its performance adequately supported by the findings presented in the article?

Yes

Is the rationale for developing the new software tool clearly explained?

Yes

Is the description of the software tool technically sound?

Yes

Are sufficient details of the code, methods and analysis (if applicable) provided to allow replication of the software development and its use by others?

Partly

Is sufficient information provided to allow interpretation of the expected output datasets and any results generated using the tool?

Yes

Reviewer Expertise:

Statistical Methods in Diagnostic Medicine, Medical AI evaluation, R Statistical Computing

I confirm that I have read this submission and believe that I have an appropriate level of expertise to confirm that it is of an acceptable scientific standard.

# Reviewer report: Reviewer response for version 1

**DOI:** 10.21956/wellcomeopenres.24690.r97069

**Published:** 2024-09-22

**Authors:** Aaron Caldwell

**Affiliations:** 1 University of Arkansas for Medical Sciences, Arkansas, USA

The authors have presented an interesting R package for comparing two diagnostic tests against a gold standard (or rather ground truth). I have no serious issues with the statistical methods, however I have significant concerns on the usability of the software in its current form. Another review has already detailed many other points. My review will detail a few issues that should be addressed to help the end user of this package and aide in the interpretation.

**Major Comments:**

 1. I think it would be preferable for users  to explicitly state which columns correspond to test1, test2 and "gold". As it currently stands, users are at the mercy of the package and have to follow a strict approach and provide a dataset with exactly 3 columns and in a particular order as well. I think it would be much more user-friendly for users to explicitly declare the columns which correspond to which test. So compareR should have three arguments: "test1", "test2", and "gold". These could be set to NULL as default and the function continue to be used like it is now. Otherwise, these could be explicitly declared and those three columns selected (even from a data frame of many columns). The code in the function could look as follows:

 compareR <- function(df,

 test1 = NULL, test2=NULL, gold = NULL,

 alpha = 0.05, margins = FALSE, multi_corr = "holm",

                      cc = TRUE, dp = 1,

                      sesp = TRUE, ppvnpv = TRUE, plrnlr = TRUE,

                      conf.int = "contemporary",

                      test.names = c("Test 1", "Test 2"), ...) {

 if( !is.null(test1) | !is.null(test2) | !is.null(gold)){

 if( is.null(test1) | is.null(test2) | is.null(gold){

 stop("At least one column named provided. Must provide other column names.")

 }

 df = df[c(test1,test2,gold)]

 }

   df <- recoder(df)

 2. The output of the functions is extremely verbose (even when passed to interpretR), and the default output for the class "compareR" is just a list. It would be useful to create & use S3 or S4 methods to simplify the output of results for users. For example, a "print" method could provide a very simple, not verbose, description of the results with arguments within the print method to allow for different items to be output to the console. A "plot" method could provide simple visualization.  Other methods could be utilized to provide other types of output (e.g., formatted tables). I think packages from the easystats group (e.g., effectsize or performance) would be good examples to get an idea of what would be better practice. I think even a report function could be used to provide a simple summary/interpretation of the results in text (see the report R package as an example).

Are the conclusions about the tool and its performance adequately supported by the findings presented in the article?

Partly

Is the rationale for developing the new software tool clearly explained?

Yes

Is the description of the software tool technically sound?

Yes

Are sufficient details of the code, methods and analysis (if applicable) provided to allow replication of the software development and its use by others?

Yes

Is sufficient information provided to allow interpretation of the expected output datasets and any results generated using the tool?

Partly

Reviewer Expertise:

Biostatistics

I confirm that I have read this submission and believe that I have an appropriate level of expertise to confirm that it is of an acceptable scientific standard, however I have significant reservations, as outlined above.

# Response

**Published:** 2024-10-01

**Authors:** Kyle Wilson

**Affiliations:** Department of Eye & Vision Science, University of Liverpool, Liverpool, England, UK

Thank you for the detailed feedback on our manuscript. It is clear from your report that you have taken the time to review not only the paper, but the code and package documentation. The effort is greatly appreciated; your suggestions have been integrated and the package is better for it. Beneath is a point-by-point response to your comments:  

1. This is an excellent point and was a definite weakness in the package. I have implemented this suggestion via a function, which gives us some flexibility to adjust testCompareR further in the future. We also felt that the users may wish to provide the data as atomic vectors and leave the df argument as NULL. This has been implemented in the new function to validate the data. It is still possible for users to supply a data frame with no indexing for the columns, but in this case testCompareR will issue a warning so that users are aware that it may introduce error.
2. Another excellent suggestion. Most users probably don’t need to access all the different levels of the list output. Some may choose to access unadjusted p values, which they still can do through usual indexing. For users who prefer a less verbose output I have provided the following three S3 methods:

summary() – this provides a title, the names of the individual tests (if given), and the contingency tables for each test against the gold standard, as well as a table of the results. It is informative and not verbose. print() – this provides a table of results. This is the bare minimum information required to review the analysis. plot() – this provides a very simple box and whisker plot for diagnostic accuracies and predictive values. We decided not to include likelihood ratios on this plot because they are not confined by the 0-1 scale and therefore compress the confidence intervals for the other metrics. Users who are particularly interested in likelihood ratios are probably more statistically savvy and therefore are likely able to plot them themselves.   Many thanks, once again, for taking a deep-dive into testCompareR. The valuable feedback has improved the manuscript and the package. If you have further comments, they will be considered and integrated. Otherwise, we hope you are satisfied with the updated manuscript.

# Reviewer report: Reviewer response for version 1

**DOI:** 10.21956/wellcomeopenres.24690.r97075

**Published:** 2024-09-11

**Authors:** Dennis Robert

**Affiliations:** 1 Director of Clinical Research (AI), Qure.ai, Bangalore, India

**SUMMARY:**

 I have read the article with great interest. The authors developed an R package titled *testCompareR *which can enable researchers to perform comparison of diagnostic accuracies of two binary diagnostic tests using paired data. Such comparisons are commonly conducted and this means that this package could be potentially very useful to many researchers. There are two similar R packages already available - *DTComPair *and *compbdt, *and the authors presented a detailed comparison of testCompareR with these already available existing two packages. testCompareR uses more statistically robust and up-to-date algorithms and has a very intuitive user interface that requires limited pre-processing steps. The article is reported in a mostly transparent manner. I congratulate the authors for developing this package as well as for reporting the details in a manuscript. However, I think the article can be improved by adding some more clarifications. Please find my list of comments below.

**COMMENTS:**

1. In the ABSTRACT, the authors quote “ *The output and efficiency of testCompareR is compared to the only other available package which performs this function, DTComPair, using a motivating example.” *This has to be modified because the comparison was not only done with *DTComPair*, but also with *compbdt*. While the latter package may not be available in CRAN, it is still available as an open-source code for anyone to use.
2. In INTRODUCTION, the authors quote: *“A newer program, compbdt, has also been published in an open-access journal. This program uses the most up-to-date statistical methods”. *As I understand, the two major motivations for the authors to develop the *testCompareR* R package is that 1) the package includes the so called “up-to-date statistical methods” and 2) *testCompareR *requires less cumbersome data pre-processing steps than *DTComPair *and *compbdt*. While it is true that the authors clearly documented the motivation, the details on what exactly are these *“up-to-date statistical methods”, *is missing. *DTComPair *uses McNemar’s test to compare sensitivity and specificity of two binary diagnostic tests in a paired study design. *testCompareR *also seems to be using the same test. Are the “up-to-date statistical methods” referring only to confidence interval construction based on Yu et.al.  or do they mean something more?. This could better be explicitly clarified in the METHODS section.
3. In Table 1, there seems to be a typo. False negative (FN) and False positive (FP) should be inter-changed so that the table reflects the correct definitions of FP (gold standard is negative, but test is positive) and FN (gold standard is positive, but test is negative).
4. In **“ *Mathematical descriptions”, *** please number the equations so that it can be easily referenced.
5. In **“ *Mathematical descriptions”, *** some of the terms in the equations are not defined. For example, what is “s”, “r”, “Se _i_”, etc. I could not find these terms in Table 3 too.  If I refer to the article by Yu et. al., it looks like "s" represents the number of diseased (positive) cases and "r" represents the number of non-diseased (negative) cases (the ratio of "s" and "s+r" will thus be the estimator of prevalence), but this should be documented in this article too to improve clarity. Please define all the terms clearly.
6. The section “Use cases” should be placed before the “Results”/”Evaluation” section. This is because the “Use cases” illustrate how the functions in the testCompareR works and it should follow the *“Using the package” *section. This will allow the users to first understand how the programming works with the functions in the package. After “Use cases”, the “RESULTS” can show the “Evaluation” results comparing with other packages.
7. Table 4 contains results with 2 decimal point precision. However, the numbers in the output under “Use cases” shows the results with single decimal point precision because you have used the default value of 1 for the argument *dp* in the function *compareR*. Please make the decimal point precision consistent between all text and tables whenever applicable.
8. Can the p values be reported in Table 4 like p < .0001, p < .001, or if p is > .001, report exact p with 2 decimal point precision? The exception to this rule is when rounding p from 3 digits to 2 digits would result in p appearing non-significant (such as p=.047).
9. It is unclear how the standard error and confidence intervals for the point estimates of diagnostic accuracy like sensitivity or specificity are obtained from DTComPair package. I assume that the function in DTComPair that is comparable with the “compare” function of “testCompareR” is “sesp.mcnemar”. However, sesp.mcnemar returns only the point estimates, test statistics, difference between point estimates and p value. The confidence interval for the difference between the point estimates are retrievable from using “sesp.diff.ci” function. But as I mentioned, it returns the CI for the difference in point estimates and not the point estimates per se. Can the authors include more details so as to make enable reproducible results? I have not tried to do this using the “compbdt” package. In fact, a fully reproducible R script that can verify the results from all three packages may be added as an Appendix/Supplement to faciliate this. I believe this code must already be available with the authors. This addition would tremendously improve the quality of the manuscript.
10. Please add a line of code “data(cass)” above the line “rbind(head(cass), tail(cass))” so that the data.frame appears in an R IDE like in Rstudio environment. This enables the users to view the data.frame easily by simply clicking the object in environment. As you have mentioned, the users of this package may be less code-savvy (like clinicians).
11. testCompareR’s compare function has an argument named “conf.int” which seems to be a fundamental argument as per the R package help document but is missing in the paper. Please include this under the list of "a range of optional parameters allow users to customise the output".
12. The three arguments – sesp, ppvnpv, plrnlr,  seems to be somewhat related. For example, if all three are FALSE, then the function will return an error. It seems at least one of them has to declared TRUE. While the error output in R console says, “No tests selected”, I think the message can be clearer; something like “No tests selected. At least one among sesp, ppvnpv and plrnlr should be declared TRUE”.
13. In DISCUSSION, the authors quote *“Despite the common use of binary diagnostic tests in medicine only one package, DTComPair, provides methods to compare the test metrics between two binary diagnostic tests using paired data”. *What about compbdt?
14. In DISCUSSION, the authors quote *“This package requires the user to be computationally literate, as several function calls are necessary to extract the outputs that would normally be published when comparing the performance of two tests”. *I agree with the authors. It is not very intuitive to use DTComPair functions, even for experienced R users. DTComPair actually requires input data to be fed in a custom (specific to DTComPair package) format of an object of class tab.paired. The ideal way would be to feed more popular and general input data class like data.frame which is what makes testCompareR very intuitive to use. Perhaps this can also be noted down in the DISCUSSION.
15. In DISCUSSION, the authors quote “ *By re-structuring the internal mechanisms, we have dramatically increased computational speed while providing additional features”. *While the authors have shown that the computational efficiency of testCompareR compared to DTComPair and compbdt packages in RESULTS, I don’t think the use of word “dramatically” is very appropriate here. Most of these function executions will only take maximum few seconds only in most scenarios, regardless of the package. I suggest toning down the language. In fact, in Figure 2B, there is considerable overlap between the distribution of computational time of testCompareR and DTComPair, except for the computation of likelihood ratios. Moreover, the main added values I see with testCompareR is its intuitive user interface (this is great, by the way!) and usage of up-to-date statistical algorithms for construction of confidence intervals with better coverage. The reduction in computational time, although true, is perhaps not that important/impactful.

Are the conclusions about the tool and its performance adequately supported by the findings presented in the article?

Yes

Is the rationale for developing the new software tool clearly explained?

Yes

Is the description of the software tool technically sound?

Yes

Are sufficient details of the code, methods and analysis (if applicable) provided to allow replication of the software development and its use by others?

Partly

Is sufficient information provided to allow interpretation of the expected output datasets and any results generated using the tool?

Yes

Reviewer Expertise:

Statistical Methods in Diagnostic Medicine, Medical AI evaluation, Real-World-Data and R Programming

I confirm that I have read this submission and believe that I have an appropriate level of expertise to confirm that it is of an acceptable scientific standard, however I have significant reservations, as outlined above.

# Response

**Published:** 2024-09-18

**Authors:** Kyle Wilson

**Affiliations:** Department of Eye & Vision Science, University of Liverpool, Liverpool, England, UK

Thank you for the detailed feedback on our manuscript. It is clear from your report that you have taken the time to review not only the paper, but the code and package documentation. The effort is greatly appreciated; your suggestions have been integrated and the package is better for it. Beneath is a point-by-point response to your comments:  

1. A line has been added to the abstract to indicate that testCompareR is evaluated against the only available package, DTComPair, and the open-source program, compbdt.
2. The statistical methods are as follows:
  - Yu et al. interval for confidence intervals of diagnostic accuracies and PVs
  - Approximation of the score method for confidence intervals for LRs (as described by Martín Andrés and Álvarez Hernández in their review - ref 11)
  - Testing the difference in diagnostic accuracies (Se and Sp) uses a complex algorithm, whereby the method is adjusted based upon prevalence and total number of particpants / samples. It seems that the ‘wall-of-text’ explaining this method is not clear in and of itself, so we have supplemented this with a diagrammatic decision tree, which better illustrates the process.
  - Testing the difference in predictive values uses a global test based on the Wald test statistic. The cause of the significance is investigated using Kosinski’s weighted generalised score statistic.
  - Testing the difference in likelihood ratios uses the Wald test considering the natural logarithm of the ratios of positive and negative likelihood ratios.
3. The typo is well-noted and has been corrected.
4. Thanks for this advice. The equations have been numbered.
5. The new version manuscript has been updated accordingly so that all terms in the equations are explicitly defined.
6. This section has now been moved as you suggest.
7. The table has been updated to maintain consistency throughout the manuscript. The exception here is the values for NLR. As these ratios are often very small, 2 significant figures were maintained. While this could be updated again at your request, it was felt that this retains important information without sacrificing consistency overall. It was also noted that somewhere in the editing process the values for PV1 were copied into PV2. They were identical. This has been updated while addressing Point 9.
8. The p values have been updated as you suggest. This makes the message clearer. Thanks.
9. You are correct that the benchmarking script already exists. This has been uploaded to the Github version of the repository and excluded from R builds (so it will not be on CRAN, in accordance with their formatting guidance) in the folder benchmarking. This Github repository has then been archived to Zenodo to maintain it for posterity. The script was cleaned up and transferred to a Quarto document, to make it more readable for interested users. The code was then re-run on a 16GB MacBook Pro with M2 Pro Silicon Chip running Ventura 13.5.1. As there were not major changes to the figures, they have not been updated, but it is nice to have seen (and to be able to demonstrate) the package functioning well on another system, also.
10. This line of code has been added.
11. This has now been updated. The conf.int argument allows users to select the Yu et al. confidence interval (“contemporary”) or the exact binomial confidence interval (“classic”) when calculating CIs for binomial proportions.
12. This is a good suggestion. This has been integrated into the code and it now fails more elegantly.
13. The discussion has been updated to reflect the availability of compbdt. We have been explicit that this is not a package, but a program.
14. Thanks again for this insightful remark. The manuscript has been updated to reflect the specifics of the data input requirements of DTComPair.
15. While the speed with which testCompareR performs is several orders of magnitude faster than compbdt, and one order of magnitude faster than DTComPair, it is very unlikely that an average user will appreciate the difference as meaningful. The 100ms processing time for DTComPair is practically instantaneous. The word dramatically has been removed.

Many thanks, once again, for taking a deep-dive into testCompareR. The valuable feedback has improved the manuscript and the package. If you have further comments, they will be considered and integrated. Otherwise, we hope you are satisfied with the updated manuscript.

# Response

**Published:** 2024-10-12

**Authors:** Dennis Robert

**Affiliations:** Qure.ai Technologies Private Limited, India

I have no further comments as all my previous comments have been adequately addressed by the author (s). The version 2 of the manuscript is a much improved version. Great work and I am sure the testCompareR will benefit a lot of researchers worldwide.
